# Acupuncture enhances fatty acid catabolism and immune modulation in children with autism

**DOI:** 10.3389/fpsyt.2025.1679154

**Published:** 2025-11-20

**Authors:** Jinbo Xu, Chao Bao

**Affiliations:** 1 The Affiliated Hospital of Nanjing University of Chinese Medicine, Nanjing, China; 2 Anhui Provincial Children's Hospital, Hefei, China

**Keywords:** autism spectrum disorder, metabolomics, proteomics, acupuncture, plasma

## Abstract

Autism spectrum disorder (ASD) is a heterogeneous neurodevelopmental condition with limited effective therapies targeting its underlying biological mechanisms. Acupuncture has shown clinical promise in improving core ASD symptoms, yet its molecular effects remain poorly understood. This study aimed to investigate the systemic mechanisms by which acupuncture exerts therapeutic effects in ASD, using an integrated plasma-based proteomic and metabolomic approach. Twenty children were enrolled, including 10 diagnosed with ASD and 10 age- and sex-matched typically developing controls. The ASD group received a 12-week standardized acupuncture intervention. Plasma samples were collected before and after treatment, and analyzed using data-independent acquisition-based LC-MS/MS for proteomics and high-resolution mass spectrometry for metabolomics. Differentially expressed proteins and metabolites were identified across healthy controls, pre-treatment, and post-treatment ASD groups, followed by pathway enrichment and integrated network analysis. Acupuncture significantly modulated biological pathways related to immune regulation, mitochondrial oxidative phosphorylation, glycolysis, folate biosynthesis, lipid metabolism, and fatty acid degradation. Notable differentially expressed proteins included CD59 and CD5L, ATP5F1A and ALDOC, and HYAL1. Metabolomic profiling revealed altered levels of lipids, lipid-like molecules, benzenoids, and folate-related metabolites, implicating changes in neurochemical balance and detoxification capacity. Fatty acid degradation pathways were also enhanced, suggesting a systemic metabolic shift toward an anti-inflammatory state. Integrating proteomic and metabolomic data, the results support a multi-system mechanism through which acupuncture restores immune–metabolic homeostasis, consistent with the holistic therapeutic framework of traditional Chinese medicine. The identification of candidate biomarkers such as CD59, ATP5F1A, and ALDOC offers potential tools for therapeutic monitoring and mechanistic insight. This study presents the first multi-omics evidence for the biological basis of acupuncture in ASD and provides a rationale for individualized interventions. These findings support future research integrating acupuncture with metabolic, nutritional, or immunological therapies. Larger-scale, controlled trials with functional validation of molecular targets are warranted to confirm these findings and guide clinical translation.

**Clinical Trial Registration:**

https://itmctr.ccebtcm.org.cn/mgt/project/view/896756278374891597/, identifier ITMCTR2025000067.

## 1 Introduction

Autism spectrum disorder (ASD) encompasses a group of complex neurodevelopmental conditions characterized by early-onset impairments in social interaction, communication, and the presence of restricted, repetitive behaviors and interests (1). The global prevalence of ASD has been increasing steadily, with recent epidemiological estimates suggesting that approximately 1 in 36 children are affected (2). Between 2017 and 2020, the United States reported a 52% rise in ASD diagnoses, reflecting heightened awareness and diagnostic capacity, but also underscoring the urgency of effective therapeutic strategies (3). Despite decades of research, ASD remains a condition with no definitive cure. Early behavioral interventions have demonstrated some efficacy in mitigating symptoms and promoting social integration (4, 5). Nonetheless, outcomes are highly variable and often insufficient in addressing core impairments such as social cognition, language development, and adaptive behavior (6). Pharmacological treatments have largely focused on managing associated symptoms like irritability or hyperactivity, while providing minimal benefit for core features of ASD and frequently causing adverse effects such as sedation, weight gain, and tremors (7, 8). These limitations, coupled with the substantial emotional and economic burden on families and healthcare systems, have intensified the search for adjunctive or alternative treatment modalities (9).

Among complementary and alternative approaches, acupuncture has gained prominence in East Asian countries, particularly China, as an adjunctive therapy for ASD. A survey in Hong Kong revealed that approximately 40% of children diagnosed with ASD received acupuncture treatment, making it the most commonly used non-conventional therapy in this population (10). Acupuncture is officially recognized in China as an alternative therapeutic option for neurodevelopmental disorders, including ASD. However, despite its widespread application, the neurobiological mechanisms through which acupuncture exerts therapeutic effects in ASD remain poorly understood. This mechanistic ambiguity limits its acceptance in mainstream clinical practice and calls for rigorous scientific inquiry.

Emerging evidence suggests that autism spectrum disorder involves a constellation of complex and systemic biological processes, including neuroinflammation, oxidative stress, synaptic dysfunction, and disturbances in gut-brain axis signaling. These multifactorial characteristics reflect the heterogeneous and dynamic nature of ASD pathophysiology, posing significant challenges for conventional single-target therapeutic strategies. Interestingly, such systemic features resonate with the holistic philosophy of traditional Chinese medicine, which emphasizes functional balance and system-level modulation across interconnected physiological networks.

In this context, multi-omics technologies offer powerful tools for decoding the biological underpinnings of acupuncture from a systems biology perspective. Proteomics allows large-scale profiling of protein expression and intracellular signaling pathways, shedding light on potential molecular targets influenced by acupuncture interventions (11). Meanwhile, metabolomics captures the dynamic flux of endogenous small-molecule metabolites associated with neurodevelopment, immune function, and energy metabolism (12). Notably, the system-level insights enabled by proteomics and metabolomics align closely with traditional Chinese medicine theory’s diagnostic and therapeutic principles, which focus on pattern differentiation and systemic regulation (13).

Despite their theoretical compatibility, integrated multi-omics approaches have rarely been applied to investigate the mechanistic basis of acupuncture in ASD. To address this critical gap, the present study employs a combined analytical strategy using data-independent acquisition (DIA)-based plasma proteomics and liquid chromatography–mass spectrometry (LC-MS)-based metabolomics. By simultaneously capturing protein-level and metabolite-level changes, this integrated approach aims to construct a comprehensive molecular profile of acupuncture’s effects in children with ASD. Ultimately, our goal is to elucidate the potential biological mechanisms through which acupuncture modulates ASD-related pathophysiology and to contribute objective evidence for its clinical application.

## 2 Materials and methods

### 2.1 Materials

The reagents and materials used in this study were sourced from the following suppliers: Dithiothreitol, iodoacetamide, ammonium bicarbonate, trifluoroacetic acid, tetraethylammonium bromide buffer, and ammonia were obtained from Sigma-Aldrich (Shanghai, China). The iRT kit as well as the Bradford protein quantification kit were purchased from Biognosys (Beijing, China). Formic acid, ultrapure water, acetonitrile, and the HighSelect™ Top14 Abundant Protein Depletion Mini Spin Columns kit were acquired from Thermo Fisher Scientific (Massachusetts, USA). Sodium dodecyl sulfate was supplied by the China National Pharmaceutical Group (Beijing, China), and mass spectrometry-grade trypsin was obtained from Promega (Beijing, China). Acetone was obtained from the Beijing Chemical Reagent Factory (Beijing, China). Sterile, single-use acupuncture needles measuring 0.30 mm in diameter and 25 mm in length were provided by the Suzhou Medical Supplies Factory (Suzhou, China).

### 2.2 Participants and groups

**Figure 1:**
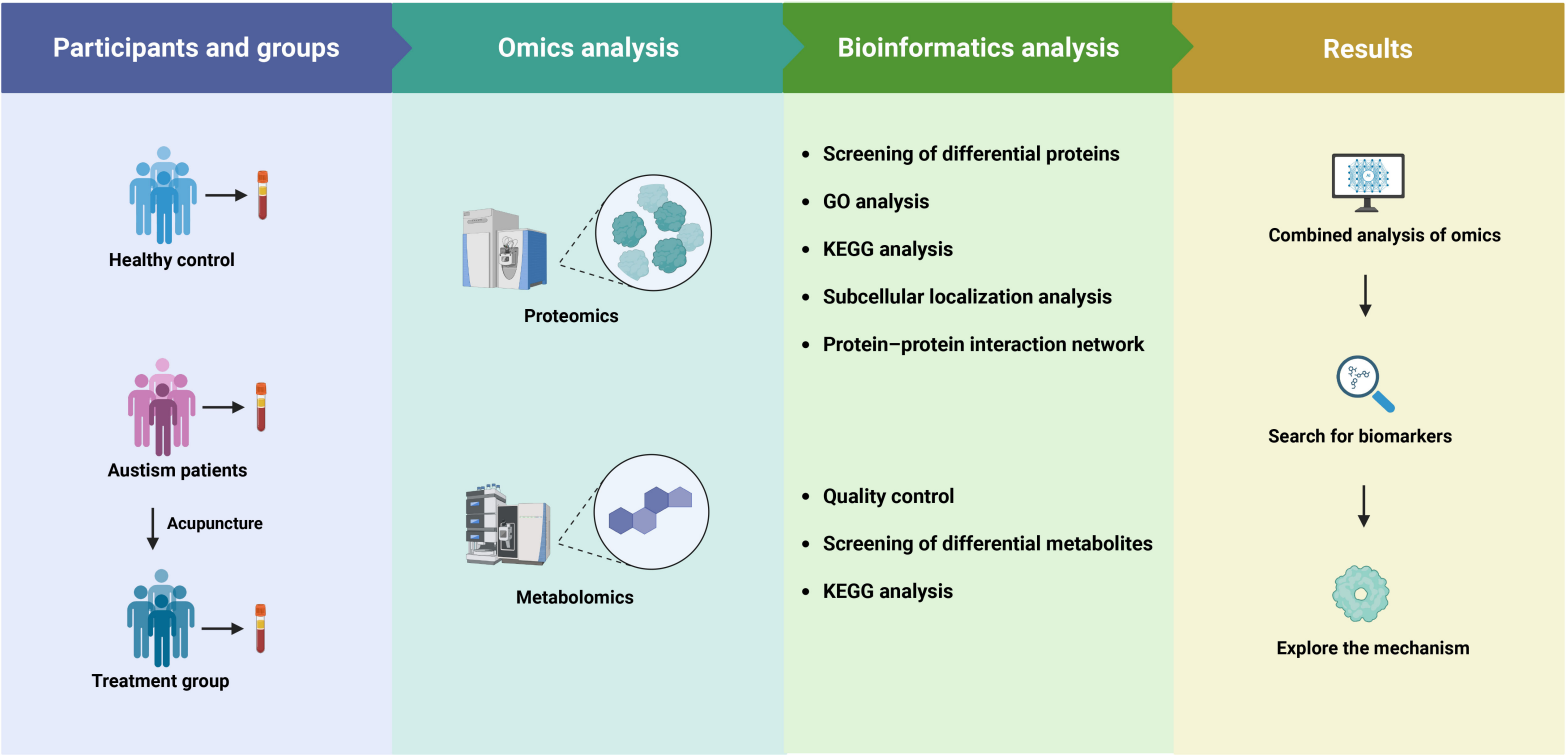
Flow diagram of the study.

This study aimed to investigate the proteomic and metabolomic changes in pediatric children with ASD undergoing a standardized acupuncture intervention, and to compare these changes with typically developing controls. A total of 20 participants were recruited from Anhui Provincial Children’s Hospital (Hefei, China), including 10 children with ASD who were clinical patients from the rehabilitation department and 10 typically developing children matched for age and sex, who were recruited from the hospital’s health examination center. The ASD group comprised 8 boys and 2 girls, aged 3 to 7 years (mean ± SD: 4.3 ± 1.1 years). The typically developing group comprised 8 boys and 2 girls, aged 3 to 7 years (mean ± SD: 4.2± 1.2 years). Inclusion criteria for ASD children included a confirmed diagnosis using the Diagnostic and Statistical Manual of Mental Disorders, Fifth Edition criteria and scoring ≥ 70 on the Childhood Autism Rating Scale. Exclusion criteria included known genetic syndromes, recent medication use, or participation in other clinical trials. The developing children had no known developmental, neurological, or psychiatric disorders and were recruited from local preschools. Peripheral venous blood (4 mL) was drawn from each participant in the morning after overnight fasting, using ethylenediaminetetraacetic acid-coated anticoagulant tubes. For ASD patients, samples were collected both before and after the acupuncture intervention; for typically developing children, a single sample was collected at baseline. Based on collection timing and clinical status, blood samples were categorized into three groups: healthy control (HC), ASD pre-treatment (ASD), and ASD post-treatment (Tx). Genomic DNA was isolated from fresh blood using the phenol–chloroform extraction technique. DNA extracts were dissolved in Tris(hydroxymethyl)aminomethane–Ethylenediaminetetraacetic acid buffer containing anhydrous ethanol and standardized to a concentration of 50 ng/μL for downstream analysis. An overview of the experimental workflow is illustrated in **Figure 1**. This study was approved by the Ethics Committee of Anhui Provincial Children’s Hospital (No. EYLL-2024-016).

### 2.3 Acupuncture treatment

The acupuncture intervention employed a standardized protocol targeting the following specific acupoints: Baihui (GV20), Sishencong (EX-HN1), Yintang (EX-HN3), Tianshu (ST25), Zusanli (ST36), and Shangjuxu (ST37). Acupoint localization was performed in accordance with the World Health Organization Standard Acupuncture Point Locations, ensuring anatomical accuracy and consistency (14). After routine disinfection of the skin, the angle and depth of needle insertion were adjusted based on the basis of the anatomical characteristics and therapeutic requirements of each acupoint. Participants underwent acupuncture three times per week, with each session lasting 30 minutes, over a 12-week treatment period. Participants in the ASD group underwent acupuncture three times per week, each session lasting 30 minutes, over a 12-week treatment period. The selection of a 12-week duration is supported by previous clinical studies in pediatric neurodevelopmental disorders, which have reported significant therapeutic effects within 12 weeks of acupuncture treatment (15, 16). All procedures were conducted in compliance with the Standards for Reporting Interventions in Clinical Trials of Acupuncture guidelines.

### 2.4 Proteomic analysis of plasma samples

#### 2.4.1 Total protein extraction

To enrich plasma proteins, 500 µg of nanomagnetic bead material (50 µL suspension volume) was aspirated and subjected to magnetic separation to discard the supernatant. The beads were washed once using an appropriate volume of wash buffer. Subsequently, an equal volume of wash buffer and plasma was added to the beads and incubated at 37°C with agitation at 1500 rpm for 1 hour. After incubation, magnetic separation was performed to remove the supernatant. The magnetic beads were washed three times using three volumes of wash buffer, shaking for 5 minutes per wash cycle. This washing procedure was repeated three times to ensure optimal removal of non-specifically bound components. The final product, bound to the surface of the magnetic beads, contained the enriched plasma proteins.

#### 2.4.2 Protein quantification and quality assessment

A bovine serum albumin standard curve was constructed following the Bradford protein assay protocol, using standard concentrations ranging from 0 to 0.5 g/L. Standard bovine serum albumin solutions and appropriately diluted sample solutions were loaded into a 96-well microplate, with a final volume of 20 µL per well. Each concentration was measured in triplicate. Next, 180 µL of G250 dye reagent was swiftly added to each well, and the plate was incubated at room temperature for 5 minutes. Absorbance was measured at 595 nm, and the standard curve was used to determine the protein concentrations of the unknown samples. For quality control, 20 μg of protein per sample was applied to a 12% SDS-PAGE gel. Electrophoresis was performed at 80 V for 20 minutes through the stacking gel, followed by 120 V for 90 minutes through the resolving gel. After electrophoresis, the protein bands were visualized using Coomassie Brilliant Blue R-250 staining, followed by destaining until clear bands were visible.

#### 2.4.3 Trypsin digestion

Each protein sample was adjusted to a final volume of 100 μL using DB lysis buffer (8 M urea, 100 mM TEAB, pH 8.5). Trypsin and 100 mM TEAB buffer were then added, and the mixture was incubated at 37°C for 4 hours, followed by a second digestion step where additional trypsin was added for overnight digestion. The digestion was halted by adjusting the pH to below 3 with formic acid, and the samples were subsequently centrifuged at 12,000 × g for 5 minutes at room temperature. The supernatant was carefully applied to a C18 desalting column, washed three times with washing buffer (0.1% formic acid, 3% acetonitrile), and eluted with elution buffer (0.1% formic acid, 70% acetonitrile). The eluates were collected and lyophilized for subsequent analysis (17).

#### 2.4.4 LC-MS/MS analysis based in DIA mode

Mobile phase A was composed of 100% H_2_O with 0.1% formic acid, while mobile phase B consisted of 80% acetonitrile and 0.1% formic acid. Lyophilized peptide samples were reconstituted in 10 μL of mobile phase A and centrifuged at 14,000 × g for 20 minutes at 4°C, and 200 ng of the resulting supernatant was used for LC-MS/MS analysis. Chromatographic separation was carried out on a Vanquish Neo ultrahigh performance liquid chromatography system, equipped with a C18 trap column (5 mm × 300 μm, 5 μm, Thermo, Cat# 174500), maintained at 50°C in a column oven. Analytical separation was performed using a C18 analytical column (PepMap™ Neo ultrahigh performance liquid chromatography, 150 μm × 15 cm, 2 μm, Thermo, Cat# ES906). Detection was conducted using a Thermo Orbitrap Astral mass spectrometer with an easy-spray electrospray ionization source. The spray voltage was set to 2.0 kV, and the ion transfer tube temperature was maintained at 290°C. Data acquisition was done in DIA mode, with the full scan MS1 range set to m/z 380–980, and the resolution was 240,000 at m/z 200, with an automatic gain control set to 500%. The precursor ion isolation window was 2 Th, and 300 DIA windows were applied. Fragmentation was performed with a normalized collision energy of 25%. MS2 spectra were acquired in the m/z 150–2000 range with a resolution of 80,000 (Astral) and a maximum injection time of 3 ms. The resulting raw files were used for downstream mass spectrometry data analysis.

#### 2.4.5 Database search and bioinformatics analysis

Protein identification and quantification were performed using ProteinPilot software version 5.0 (AB Sciex), with the following parameters: enzyme specificity was set to trypsin; minimum peptide length was set to 7 amino acids; fixed modification was carbamidomethylation of cysteine residues; variable modification included methionine oxidation; and the false discovery rate threshold was controlled at 1%. The UniProtKB/Swiss-Prot human protein database was used as the reference for database searching. The quantitative data were uploaded to the OMICSBEAN platform for subsequent statistical and functional analysis. Data normalization was carried out, followed by two-tailed t-tests to identify differentially expressed proteins (DEPs). A fold change threshold of >1.2 for upregulation and <0.83 for downregulation was applied, with statistical significance defined as FDR-adjusted p < 0.05. Principal component analysis (PCA) was conducted using MetaboAnalyst 5.0, and partial least squares discriminant analysis (PLS-DA) was performed via SIMCA-P version 14.1 (Sartorius Stedim Data Analytics AB, Umea, Sweden). The PLS-DA model was validated through 7-fold cross-validation and tested with 200 random permutation cycles. Identified DEPs were further analyzed through volcano plot visualization, hierarchical cluster heatmaps (18), and functional enrichment using Gene Ontology (GO) and Kyoto Encyclopedia of Genes and Genomes (KEGG) pathway analyses (19).

### 2.5 Metabolomics analysis of plasma samples

#### 2.5.1 Plasma pretreatment and mass spectrometry analysis

To prepare the metabolite extraction solution, the internal standard L-2-chlorophenylalanine (0.3 mg/mL in methanol) was first mixed with a prechilled solvent consisting of methanol and acetonitrile (2:1, v/v) at −20 °C. Each plasma sample (100 μL) was then combined with 300 μL of the prepared extraction solvent, followed by sonication in an ice-cooled water bath for 10 minutes. The mixture was subsequently incubated at −20 °C for 30 minutes and centrifuged at 13,000 × g for 15 minutes at 4 °C. From the resulting supernatant, 100 μL was transferred into a clean autosampler vial, and 10 μL from each sample was pooled to create a quality control mixture. Metabolic profiling was performed using a Waters ACQUITY UPLC system coupled with a high-resolution Q-TOF Synapt G2 mass spectrometer (Waters, USA). Chromatographic separation was achieved on an ACQUITY UPLC BEH C18 column (2.1 × 100 mm, 1.7 μm particle size) maintained at 45 °C. The mobile phases were composed of 0.1% formic acid in water (mobile phase A) and 0.1% formic acid in acetonitrile (mobile phase B), delivered at a flow rate of 0.4 mL/min. The sample injection volume was 2 μL. Mass spectrometric detection was performed using an electrospray ionization source, with data collected in both positive and negative ionization modes.

#### 2.5.2 Processing of the results of metabolomics mass spectrometry

Raw spectral data were processed using Progenesis QI 2.0 (Nonlinear Dynamics, Newcastle, UK) for peak detection, alignment, and normalization. Compound annotation employed publicly available spectral libraries including MassBank, HMDB, LipidBlast and METLIN; redundant identifications were removed based on scoring criteria and retention time consistency. Subsequent statistical analyses were performed in R using the metaX package (v2.x).

#### 2.5.3 Metabolomics data analysis

The metabolomics data profile was analyzed using multivariate statistical methods. PCA and PLS-DA were conducted. The variable importance (VIP) score from PLS-DA was obtained. Metabolites with VIP scores ≥ 1, fold change ≥ 1.5 or ≤ 0.83, and p < 0.05 were identified as differentially expressed metabolites (DEMs). Pathway analysis was performed using MetaboAnalyst 5.0.

## 3 Results

### 3.1 DIA proteomics analysis

**Figure 2:**
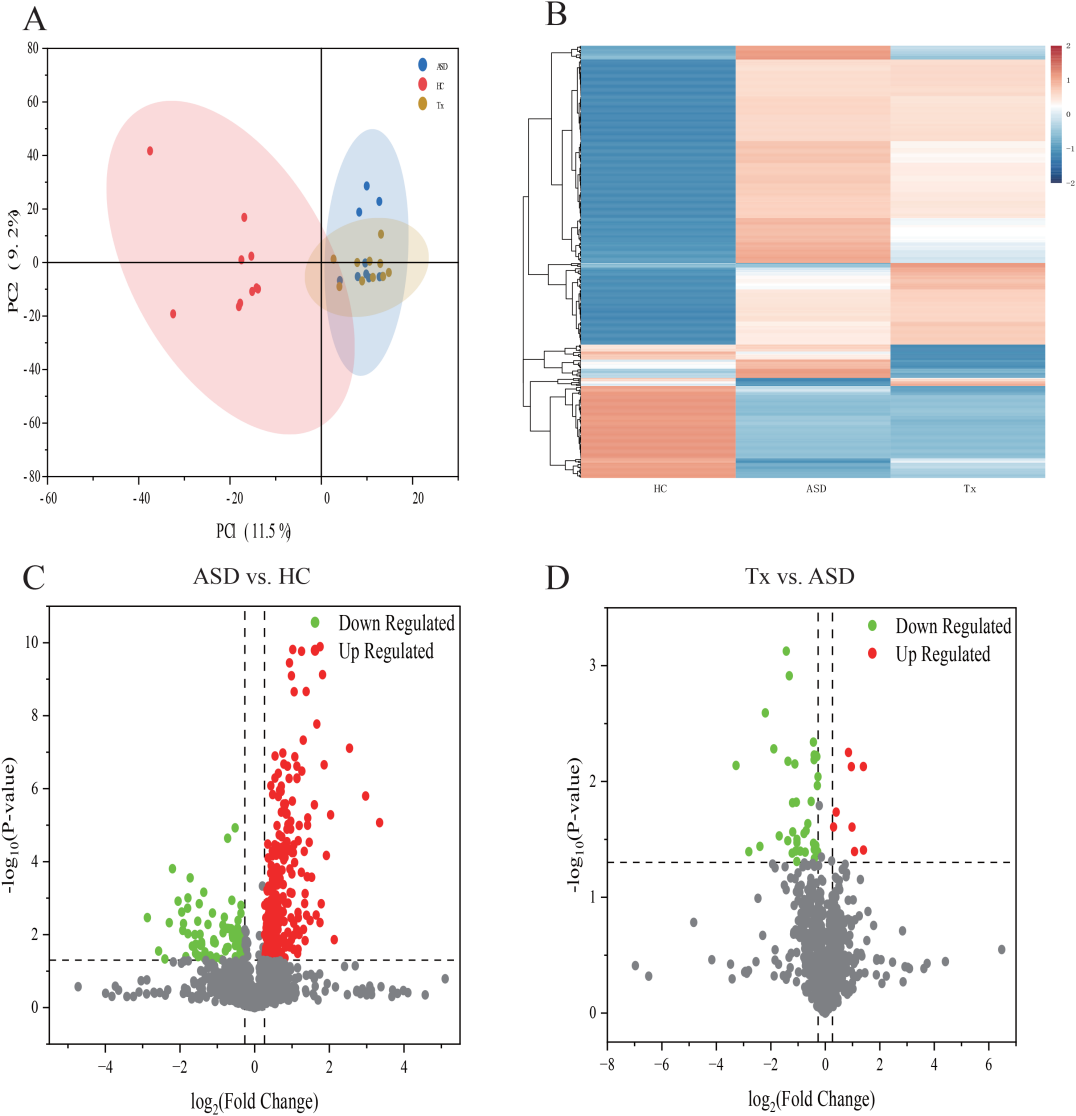
Statistical analysis of plasma proteins detected by proteomics. **(A)** PCA analysis of plasma proteins among HC, ASD, Tx groups; **(B)** Cluster map of the differentially expressed proteins among HC, ASD, Tx groups; **(C)** Volcano plots of the differentially expressed proteins between HC and ASD groups; **(D)** Volcano plots of the differentially expressed proteins between ASD and Tx groups.

To identify significant DEPs among the HC, ASD and Tx groups, DIA proteomics was employed to quantify protein expression levels across all samples. The PCA of protein quantification results is presented in **Figure 2A**, where a higher degree of clustering among replicate samples indicates better reproducibility of the quantification data. DEPs were defined based on the criteria of fold change ≥ 1.2 and p-value < 0.05. Differential expression between the ASD vs. HC and Tx vs. ASD groups was visualized through heatmaps (**Figure 2B**) and volcano plots (**Figures 2C, D**). Compared with the HCs, the ASD group presented 256 upregulated and 84 downregulated DEPs. Furthermore, in comparison to the ASD group, the Tx group showed 8 upregulated and 38 downregulated DEPs. Specifically, the expression levels of ALDOA, RPLP2, ALDOC, UBB, SGRN, RNH1, HSPA6, TFF3, HYAL1, COL19A1, and PRAM1 were elevated in the ASD group relative to those in the HC group, whereas the expression of these proteins was reduced following treatment in the Tx group. Conversely, LDHC levels were lower in ASD subjects than in HCs, but increased following acupuncture treatment in the Tx group (**Supplementary Table 1**).

**Figure 3:**
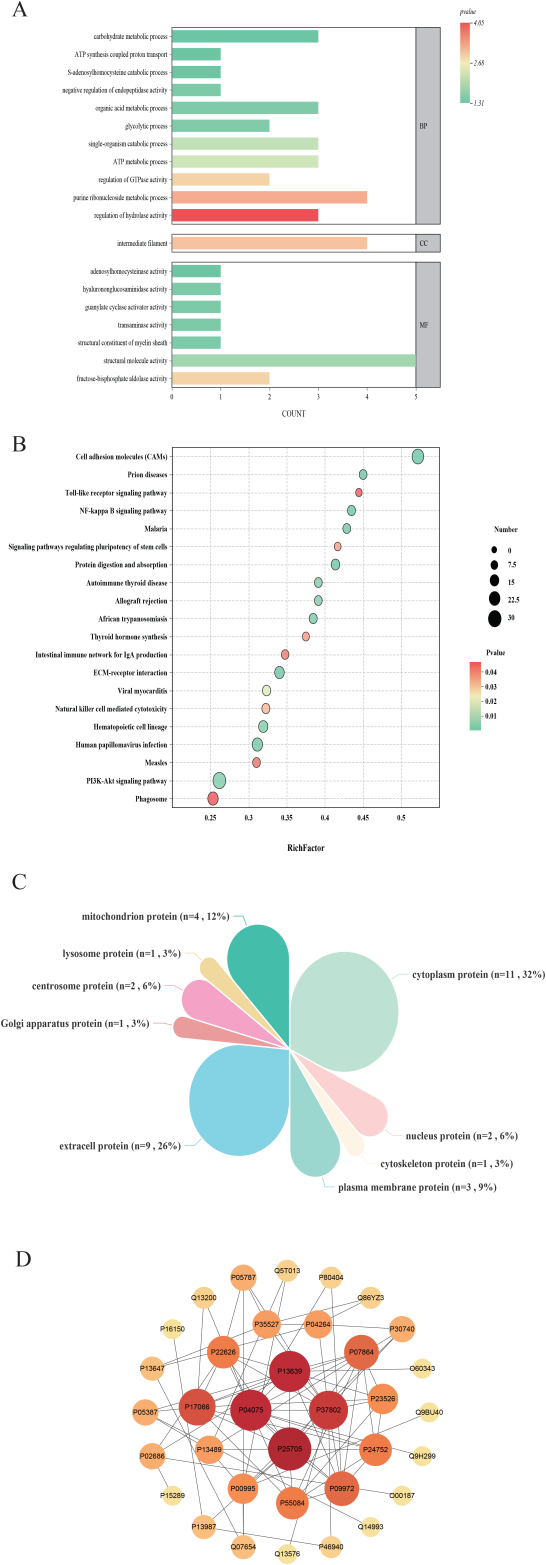
Comparison of differentially expressed proteins of plasma. **(A)** GO enrichment analysis of differentially expressed proteins between ASD and Tx groups; **(B)** KEGG pathway analysis of differentially expressed proteins between ASD and Tx groups; **(C)** Subcellular localization analysis of differentially expressed proteins between ASD and Tx groups; **(D)** Protein-protein interaction network analysis of differentially expressed proteins between ASD and Tx groups.

We focused our analysis on the DEPs between the Tx and ASD groups, and subsequently performed functional enrichment analyses. GO enrichment revealed that “regulation of hydrolase activity” was the most significantly enriched term in the biological process (BP), “intermediate filament” under cellular component (CC), and “fructose-bisphosphate aldolase activity” under molecular function (MF) categories (**Figure 3A**). Additionally, KEGG pathway analysis showed that DEPs were significantly enriched in the “cell adhesion molecules (CAMs)”, “Prion diseases”, “Toll-like receptor signaling pathway”, “NF-kappa B signaling pathway”, and “Malaria” pathways (**Figure 3B**). We further employed WoLF PSORT software to predict the subcellular localization of these DEPs, followed by categorical statistical analysis. Among the DEPs between the ASD and Tx groups, 32% were localized to the cytoplasm, 26% to the extracellular region, 12% to the mitochondrion, 9% to the plasma membrane, 6% to the nucleus, 6% to the centrosome, and 3% each to the lysosome, Golgi apparatus, and cytoskeleton (**Figure 3C**). To explore the molecular mechanism underlying acupuncture intervention, we extracted protein interaction data for the potential target proteins and constructed a protein-protein interaction (PPI) network (**Figure 3D**). Within this network, the hub proteins with the highest degree of connectivity included EEF2 (P13639), ATP5F1A (P25705), ALDOA (P04075), and TAGLN2 (P37802). These findings suggest that these central proteins may serve as key molecular targets of acupuncture therapy in ASD.

### 3.2 LC-MS of metabolic profiles

**Figure 4:**
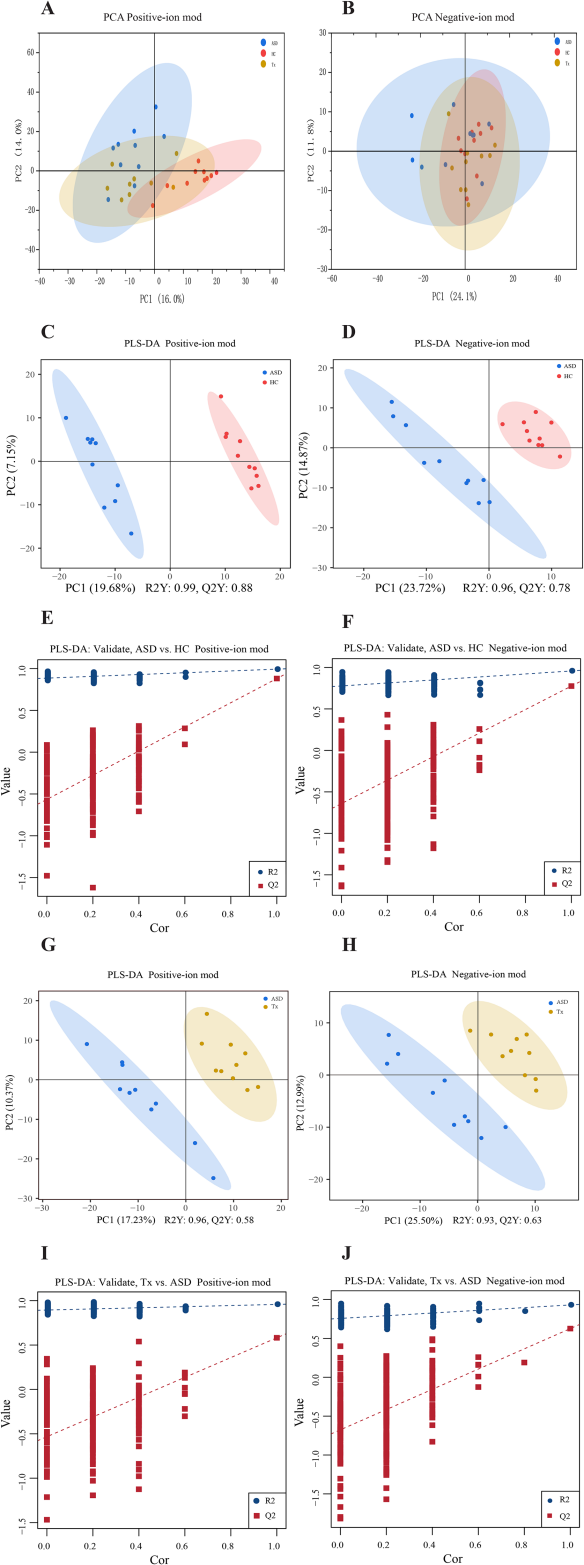
Statistical analysis of plasma metabolites detected by metabolomics in positive and negative-ion modes. **(A, B)** PCA analysis of plasma metabolites among HC, ASD, and Tx groups in positive and negative-ion modes; **(C, D)** PLS-DA score plots between HC and ASD groups in positive and negative-ion modes; **(E, F)** PLS-DA validate plots between HC and ASD groups in positive and negative-ion modes; **(G, H)** PLS-DA score plots between ASD and Tx groups in positive and negative-ion modes; **(I, J)** PLS-DA validate plots between ASD and Tx groups in positive and negative-ion modes.

Ultra-performance liquid chromatography coupled with quadrupole time-of-flight mass spectrometry was utilized to compare plasma metabolic profiles across the three groups. Initially, PCA was conducted to provide an overview of the global metabolic landscape in both positive-ion and negative-ion modes. Distinct separation of the HC, ASD, and Tx groups was observed in both modes (**Figures 4A, B**), indicating substantial differences in endogenous plasma metabolites. To further explore metabolic alterations between groups, PLS-DA was applied. As illustrated in **Figures 4C, D**, the metabolic trajectories of the HC and ASD groups were well separated with minimal overlap, suggesting pronounced biological alterations associated with ASD. These findings demonstrate that metabolomics is capable of effectively distinguishing physiological and pathological states. Moreover, the ASD group displayed tight clustering, reflecting low intragroup variability. After acupuncture treatment, significant differences were observed in the metabolic trajectories between the ASD and Tx groups, indicating that acupuncture treatment can affect the plasma metabolic profile of children with ASD (**Figures 4G, H**). The predictive performance and robustness of the PLS-DA models were evaluated through cross-validation. The results indicate that the PLS-DA models exhibit strong predictive ability and minimal risk of overfitting in both positive-ion mode (**Figures 4E, I**) and negative-ion mode (**Figures 4F, J**).

**Figure 5:**
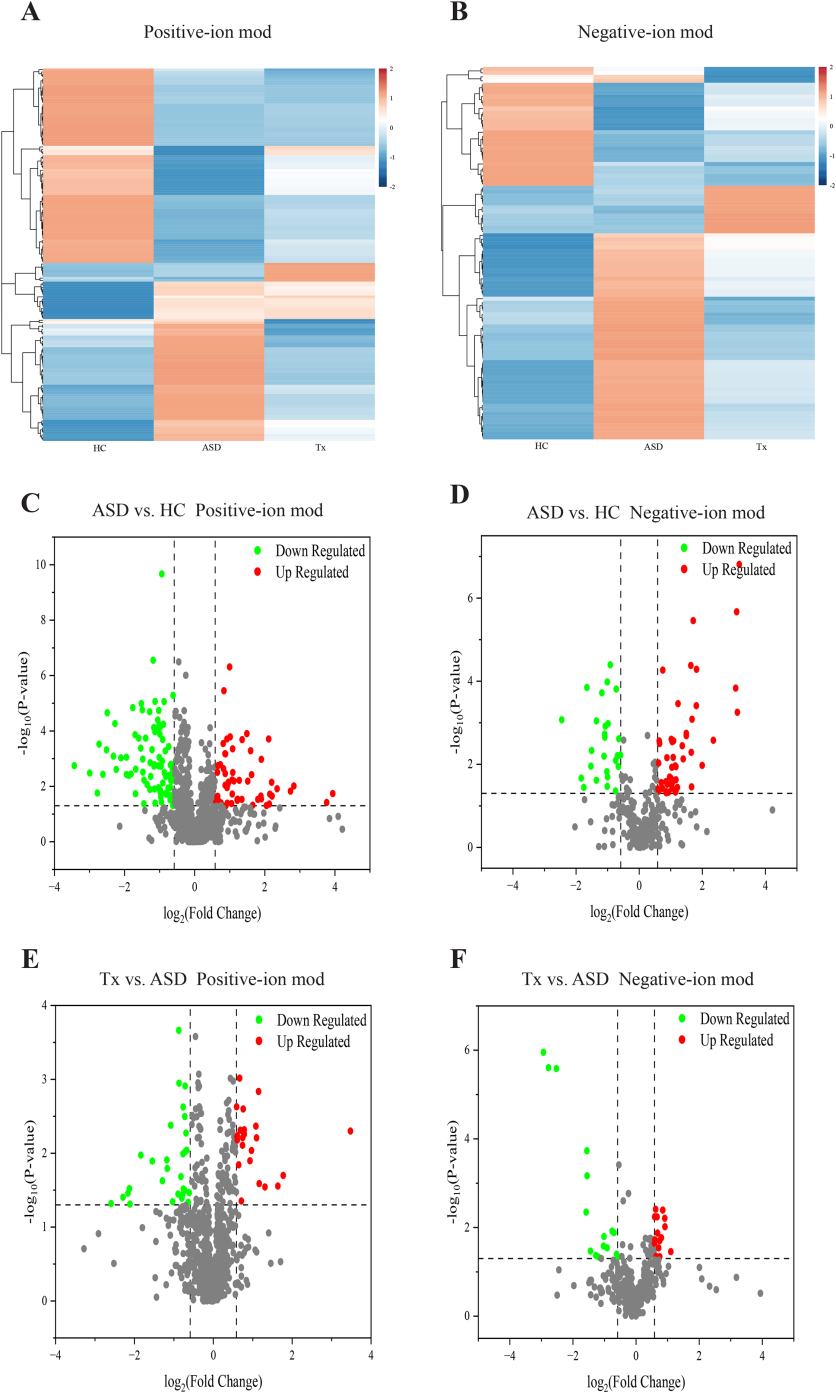
Comparison of differentially expressed metabolites of plasma. **(A, B)** Cluster analysis of differentially expressed metabolites among HC, ASD, and Tx groups in positive and negative-ion modes; **(C, D)** Volcano plots of differentially expressed metabolites between HC and ASD groups in positive and negative-ion modes; **(E, F)** Volcano plots of differentially expressed metabolites between ASD and Tx groups in positive and negative-ion modes.

Heatmaps generated using R software (v. 3.4.1) were employed to compare the average normalized abundance of differentially expressed metabolites among the three groups, demonstrating marked variations in metabolite profiles across groups (**Figures 4A, B**). Each color block in the heatmap reflects the relative abundance of a specific metabolite based on normalization, with rows representing individual metabolites and columns corresponding to the HC, ASD, or Tx group. Color intensity indicates the relative metabolite level, thereby allowing intuitive visualization of expression patterns. The metabolite content in the HC and ASD groups exhibited clearly differed, and the levels of several potential biomarkers were also distinctly different between the Tx and ASD groups. Importantly, metabolite levels in the Tx group tended to approximate those in the HC group, suggesting a partial reversal of ASD-related metabolic alterations. To identify discriminatory metabolites, variable importance in VIP values from PLS-DA were used. Metabolites with VIP > 1.0 and p < 0.05 were designated as potential biomarkers. In positive-ion mode, 137 metabolites (54 upregulated, 83 downregulated) were identified between ASD and HC group, and 54 metabolites (25 upregulated, 29 downregulated) between Tx and ASD group. Similarly, in negative-ion mode, 74 (48 upregulated, 26 downregulated) and 28 (15 upregulated, 13 downregulated) differential metabolites were identified, respectively. Details of these metabolites are provided in **Supplementary Table 2**. VIP plots (**Figures 5C–F**) illustrate that ion fragments located closer to the extremes of the V-shaped distribution contributed more significantly to the observed metabolic shifts, whereas those near the center have lesser influence.

In positive-ion mode, the ASD group exhibited elevated levels of (R)-isomucronulatol, oleamide, morusimic acid D, linoleic acid amide, osthole, cassipourol, cis-11,14-eicosadienoic acid, cohibin C, 2-amino-1,3,4-tetradecanetriol, toyocamycin, Arg-Phe-Ala, 3-hydroxyundecanoic acid, denticulatin B, and phytosphingosine compared to HC. Notably, these metabolites were reduced in the Tx group, indicating a trend toward normalization. Conversely, 3,7,8,15-Scirpenetetrol, garcinone C, metesind, myxopyronin B, eplerenone, (Ethoxymethyl)benzene, dehydrocarvacrol, angelitriol, magnoshinin, hymenoflorin, (S)-(E)-2’-(3,6-Dimethyl-2-heptenyl)-3’,4’,7-trihydroxyflavanone, ethyl 6,7-dimethoxy-4-oxo-2,3-dihydro-1H-naphthalene-2-carboxylate, praeruptorin E, dibenzylamine, nicotine-cis-N-oxide, biotin-XX hydrazide, and didesmethylisoproturon were reduced in ASD patients relative to HCs but increased following acupuncture intervention. In negative-ion mode, metabolites such as perfluoroheptanoic acid, palifosfamide, heptafluorobutyric acid, perfluorohexanoic acid, and dibutylone were elevated in ASD, while bellidifolin, betulalbuside A, and lumpidin were diminished. Acupuncture appeared to reverse these trends, aligning the Tx group’s metabolite levels more closely with the HC group.

**Figure 6:**
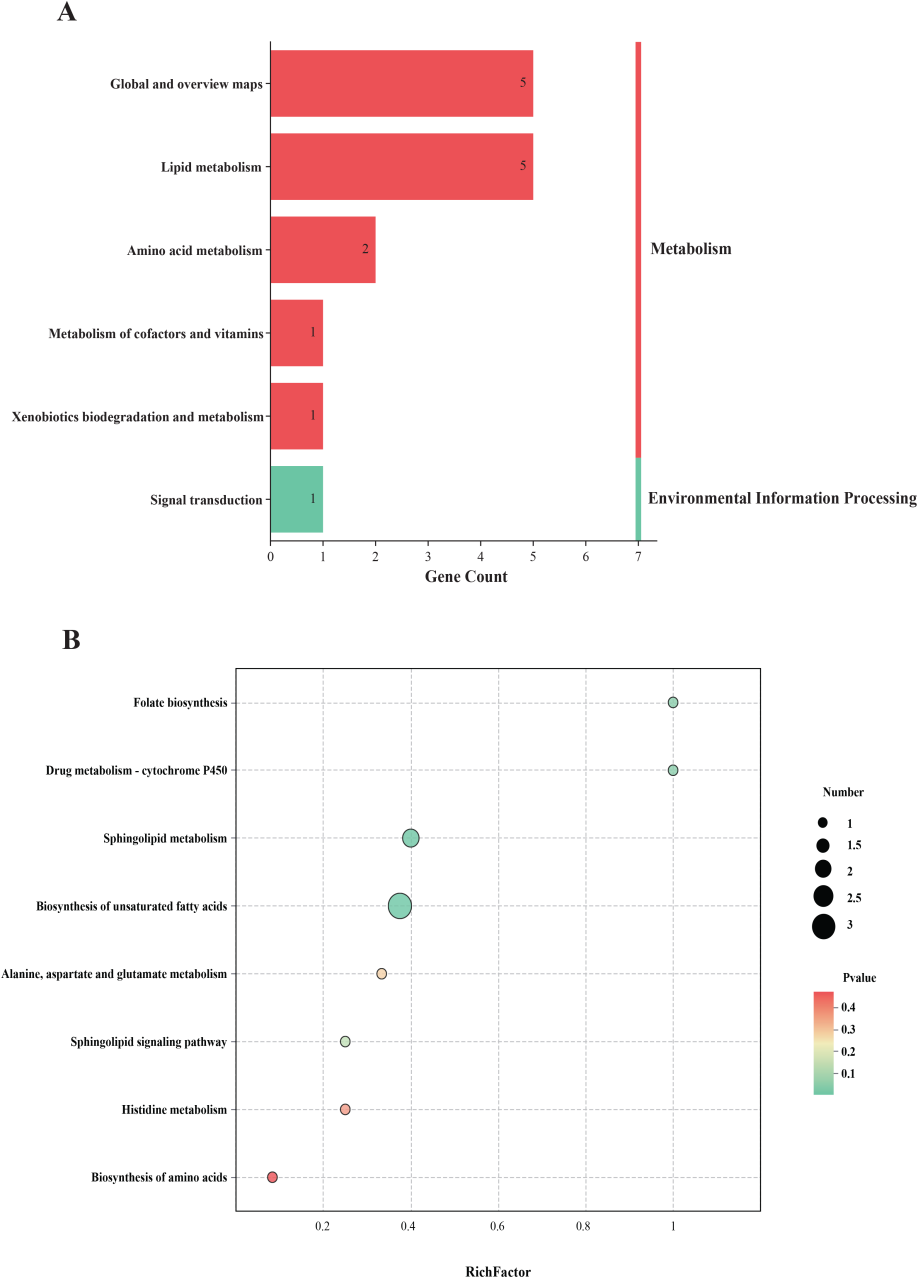
The biological analysis of differentially expressed metabolites of plasma. **(A)** KEGG classification of differentially expressed metabolites; **(B)** KEGG pathway analysis of differentially expressed metabolites.

In total, 82 differentially expressed metabolites were identified between the Tx and ASD groups. According to KEGG annotations, these metabolites were predominantly associated with lipid metabolism, amino acid metabolism, and global metabolic pathways (**Figure 6A**). Further analysis using MetaboAnalyst 5.0 was conducted to elucidate potential mechanisms underlying the effect of acupuncture on ASD. Pathways with an impact score > 0.10 were selected as candidate targets. As shown in **Figure 6B**, key pathways potentially modulated by acupuncture included folate biosynthesis, cytochrome P450-mediated drug metabolism, sphingolipid metabolism, biosynthesis of unsaturated fatty acids, and alanine, aspartate, and glutamate metabolism.

### 3.3 Combined analysis of proteomics and metabolomics

**Figure 7:**
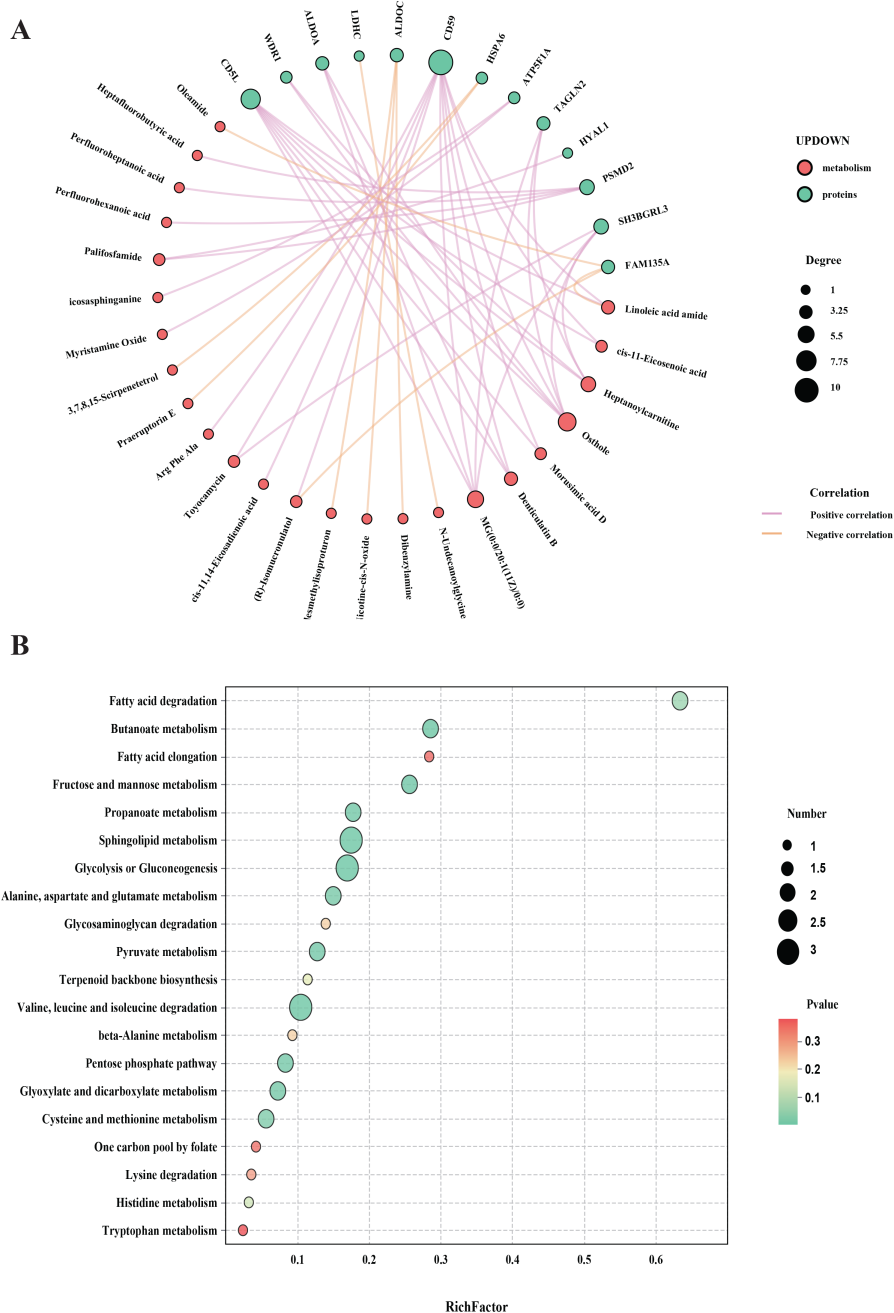
Combined analysis of proteomics and metabolomics. **(A)** Network diagram of the association analysis of proteins and metabolites; **(B)** Joint pathway analysis diagram of proteomics and metabolomics.

To further elucidate the mechanism of acupuncture in treating ASD, we constructed an integrated interaction network combining proteomics and metabolomics. A total of 46 DEPs and 82 DEMs identified from the above analyses were imported into KEGG Mapper for network correlation analysis. The resulting protein-metabolite interaction network, built with high-confidence associations. Among the key metabolites identified were osthole, MG(0:0/20:1(11Z)/0:0), heptanoylcarnitine, linoleic acid amide, and palifosfamide. These metabolites were enriched in pathways such as fatty acid degradation, butanoate metabolism, fatty acid elongation, fructose and mannose metabolism, and propanoate metabolism, all of which are significantly modulated by acupuncture. These metabolic pathways are closely associated with several core regulatory proteins, including CD59, CD5L, SH3BGRL3, and PSMD2, which may serve as potential therapeutic targets in acupuncture-mediated ASD intervention (**Figures 7A, B**).

## 4 Discussion

ASD presents a multifaceted clinical and biological challenge, with its pathogenesis involving immune dysregulation, mitochondrial dysfunction, synaptic abnormalities, and environmental influences. Despite the widespread use of acupuncture in clinical practice for ASD in East Asia, the molecular mechanisms underlying its therapeutic effects have remained poorly understood (20). This study represents the first attempt to systematically integrate proteomic and metabolomic approaches to elucidate the systemic impact of acupuncture in children with ASD. By analyzing paired pre- and post-treatment samples, and including age- and sex-matched typically developing controls, our findings shed light on key molecular networks modulated by acupuncture and reveal potential biomarkers and therapeutic targets.

One of the most prominent findings of this study was the restoration of several immune-related proteins following acupuncture, notably CD59, CD5L, HSPA6, and EEF2. These proteins converge on pathways involved in innate immunity, complement regulation, and neuroinflammation, which are increasingly recognized as central to ASD pathophysiology (21–25). Our results support previous observations that complement system disruption impairs synaptic pruning during neurodevelopment, leading to cortical hyperconnectivity, a hallmark of ASD (26, 27). More importantly, we propose that acupuncture may exert its therapeutic effects through targeted modulation of the complement cascade and associated inflammatory mediators, thus promoting immunological homeostasis. This immune-modulatory role of acupuncture aligns with accumulating evidence linking systemic inflammation and neuroimmune signaling in ASD (28, 29). Notably, CD59 and CD5L are key regulators of complement-mediated cytotoxicity, and their upregulation post-treatment may reflect a shift toward resolving chronic immune activation. These findings offer a potential mechanistic explanation for the clinical improvements observed with acupuncture and highlight the immune system as a viable intervention target in ASD.

In parallel with immune changes, proteomic profiling revealed significant alterations in mitochondrial and glycolytic enzymes following acupuncture. These included ATP5F1A, ALDOA, ALDOC, LDHC, and HYAL1. These differentially expressed proteins play key roles in energy metabolism: ATP5F1A is involved in mitochondrial oxidative phosphorylation; ALDOA and ALDOC function in glycolysis; LDHC catalyzes the conversion of lactate to pyruvate during anaerobic glycolysis; and HYAL1 mediates the degradation of hyaluronan, contributing to extracellular matrix and glycosaminoglycan metabolism (30–33). Mitochondrial dysfunction is a consistent feature in ASD, and its impact on neuronal function, synaptic transmission, and neurodevelopment is well-documented (34–37). Our findings suggest that acupuncture may contribute to restoring mitochondrial homeostasis and energy supply in the autistic brain. Interestingly, ALDOC, a brain-specific isoform expressed in hippocampal and cerebellar regions, was among the most responsive proteins to treatment, supporting a regionally selective effect. The observed upregulation of ATP5F1A further implies enhanced oxidative phosphorylation capacity post-treatment. These data collectively suggest that acupuncture may rebalance central energy metabolism, offering both neuroprotective and functional support in ASD-affected neural circuits.

Metabolomic analysis revealed that plasma metabolites regulated by acupuncture treatment in children with ASD were primarily involved in lipids and lipid-like molecules (such as betulalbuside A, lumpidin, and eplerenone) and benzenoids (such as dibutylone, dibenzylamine, and didesmethylisoproturon). The brain’s high lipid content and dependency make it especially sensitive to lipid imbalances, which are closely linked to synaptic function and myelin integrity (38). Our results suggest that acupuncture may help restore lipid homeostasis, potentially benefiting neurodevelopmental processes affected in ASD (39–41). Meanwhile, acupuncture also modulated plasma levels of benzenoid compounds, a class of environmental neurotoxicants. Benzene derivatives have been shown to induce structural and functional brain damage, including myelin abnormalities and axonal loss (42). Their reduction following treatment may reflect enhanced detoxification or metabolic clearance. Considering the established association between early-life benzene exposure and ASD risk (43), as well as links to ADHD-like symptoms (44), this suggests acupuncture may play a dual role in regulating both endogenous lipid metabolism and exogenous toxin burden, thereby supporting neurobiological function in ASD.

Another salient pathway enriched in our analysis was folate biosynthesis. Folate is a key cofactor in one-carbon metabolism, which governs essential biological processes such as DNA methylation, nucleotide synthesis, and redox balance (45–49). Dysregulation or deficiency of folate has been implicated in ASD, particularly through epigenetic mechanisms like hypomethylation. Our findings suggest that acupuncture may support folate metabolism (50–53), potentially enhancing one-carbon cycle activity and contributing to improved neurodevelopmental outcomes (54, 55). This is especially relevant given the consistently reported low serum folate levels in individuals with ASD and the well-established benefits of maternal folate supplementation in reducing ASD risk (56–60). Acupuncture may thus serve as a non-pharmacological approach to improve methylation capacity and mitigate neurodevelopmental vulnerability in at-risk populations.

Our study also revealed significant modulation of fatty acid degradation pathways, suggesting that acupuncture may promote a shift toward anti-inflammatory lipid mediator profiles. Polyunsaturated fatty acids, particularly arachidonic acid derivatives, play key roles in immune regulation (61). In ASD, an elevated omega-6 to omega-3 fatty acid ratio is commonly observed and is associated with a heightened proinflammatory state (62). Notably, increased levels of arachidonic acid-derived diols have been linked to more severe ASD symptoms and impaired adaptive functioning (63–65). These diols are produced from anti-inflammatory epoxy fatty acids via soluble epoxide hydrolase, a conversion that diminishes anti-inflammatory potential (66, 67). Our results suggest that acupuncture may inhibit this enzymatic conversion, thereby preserving epoxy fatty acids, which are known to suppress NF-κB activation and proinflammatory cytokine transcription (66). This shift may also enhance IL-10 production in microglia, a key anti-inflammatory and neuroprotective mechanism within the central nervous system (68). Taken together, these findings offer a novel perspective on acupuncture’s immuno-lipid effects, highlighting a potentially synergistic mechanism that bridges energy metabolism, lipid signaling, and immune modulation in ASD.

Collectively, our results suggest that acupuncture exerts therapeutic effects in ASD by restoring system-level homeostasis through a multi-pathway mechanism. These mechanisms include immune modulation, mitochondrial support, synaptic regulation, epigenetic enhancement, and detoxification—representing a holistic correction of biological imbalances consistent with traditional Chinese medicine theory. This multi-omics study also identifies novel biomarker candidates (such as CD59, ATP5F1A, ALDOC, LDHC, HYAL1) for tracking acupuncture response and provides a biological basis for individualized treatment approaches. Furthermore, our findings open avenues for integrating acupuncture with dietary or metabolic therapies to enhance clinical efficacy.

However, several limitations should be acknowledged. First, the small sample size may reduce statistical power and generalizability. Recruitment was challenging due to the vulnerable nature of children with ASD and parental hesitancy toward invasive procedures such as blood sampling. In addition, the high costs and technical demands associated with proteomic and metabolomic analyses further constrained sample size. Second, the absence of a sham acupuncture control group limits the ability to isolate the specific physiological effects of acupuncture. It is ethically controversial to expose pediatric participants to invasive procedures that offer no direct therapeutic benefit, and many caregivers were understandably reluctant to consent to non-therapeutic interventions. Furthermore, true and sham acupuncture differ in tactile perception, which may be easily detected by children with heightened sensory sensitivity, thus complicating blinding procedures. Future research should aim to overcome these challenges by recruiting larger and more diverse populations, and by developing ethically appropriate and methodologically feasible control conditions to improve the rigor and interpretability of the findings.

Although several key DEPs and DEMs were identified in this study, their biological functions remain to be experimentally verified. Future investigations integrating targeted assays such as enzyme-linked immunosorbent assays and western blotting will be essential to validate these candidate biomarkers and to strengthen the mechanistic interpretation of the omics results. Furthermore, to enhance the translational impact of our study, future research should aim to correlate these molecular changes with clinically relevant behavioral outcomes in children with ASD. This could be achieved through the inclusion of standardized behavioral assessments, such as the ABC or CARS, before and after treatment. By examining the relationship between specific molecular alterations and improvements in ASD symptoms, we can provide a more comprehensive understanding of the therapeutic mechanisms of acupuncture. Such studies will help bridge the gap between molecular findings and clinical outcomes, ultimately informing more personalized and effective treatments for children with ASD.

## 5 Conclusion

**Figure 8:**
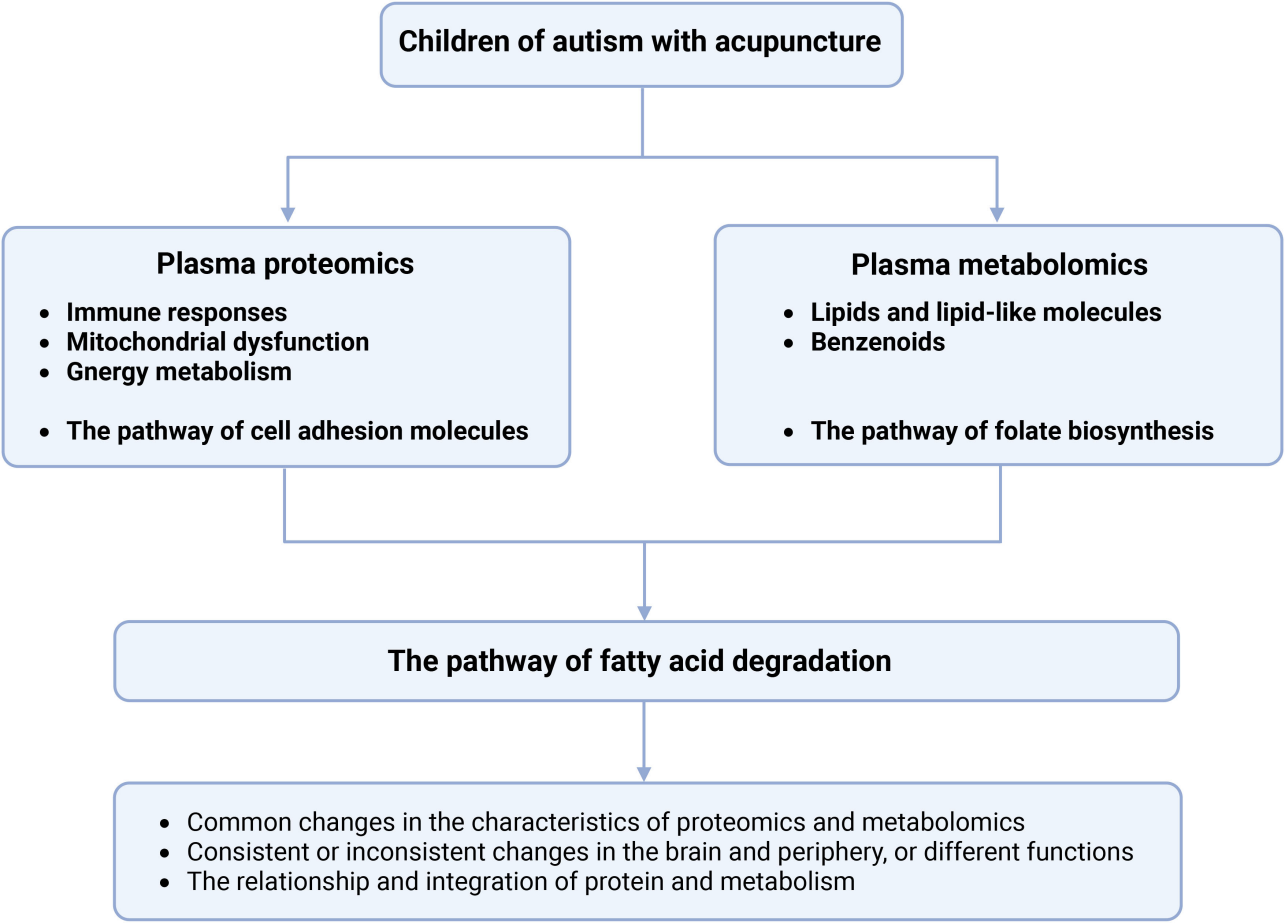
The mechanisms and potential biomarkers can be obtained in children with ASD with acupuncture treatment. Bold letters indicate that these pathways are related to the DEPs and DEMs in this study.

This study provides the first multi-omics evidence that acupuncture exerts therapeutic effects in children with ASD by modulating immune, metabolic, neurochemical, and detoxification pathways (**Figure 8**). Key proteomic changes included the regulation of immune markers, mitochondrial and glycolytic proteins, and synaptic-related molecules. Metabolomic profiling revealed disruptions in lipids, benzenoids, and folate biosynthesis. Importantly, acupuncture appeared to enhance fatty acid degradation, suggesting a shift toward anti-inflammatory lipid signaling. These findings support a systems-level mechanism through which acupuncture restores physiological balance, aligning with Traditional Chinese Medicine principles. Candidate biomarkers identified in this study may inform therapeutic monitoring and individualized interventions. Further studies with larger samples and functional validation are needed to confirm these mechanisms and support clinical translation.

## Data Availability

The raw data supporting the conclusions of this article will be made available by the authors, without undue reservation.

## References

[1] Frye RE . Social skills deficits in autism spectrum disorder: potential biological origins and progress in developing therapeutic agents. CNS Drugs. (2018) 32:713–34. doi: 10.1007/s40263-018-0556-Y, PMID: 30105528 PMC6105175

[2] Maenner MJ Warren Z Williams AR Amoakohene E Bakian AV Bilder DA . Prevalence and characteristics of autism spectrum disorder among children aged 8 years – Autism and Developmental Disabilities Monitoring Network, 11 sites, United States, 2020. MMWR Surveill Summ. (2023) 72:1–14. doi: 10.15585/mmwr.ss7202a1, PMID: 36952288 PMC10042614

[3] Li Q Li Y Liu B Chen Q Xing X Xu G . Prevalence of autism spectrum disorder among children and adolescents in the United States from 2019 to 2020. JAMA Pediatr. (2022) 176:943–5. doi: 10.1001/jamapediatrics.2022.1846, PMID: 35789247 PMC9257681

[4] Chen L Shi XJ Liu H Mao X Gui LN Wang H . Oxidative stress marker aberrations in children with autism spectrum disorder: a systematic review and meta-analysis of 87 studies (N = 9109). Transl Psychiatry. (2021) 11:15. doi: 10.1038/s41398-020-01135-3, PMID: 33414386 PMC7791110

[5] Eldevik S Hastings RP Hughes JC Jahr E Eikeseth S Cross S . Meta-analysis of early intensive behavioral intervention for children with autism. J Clin Child Adolesc Psychol. (2009) 38:439–50. doi: 10.1080/15374410902851739, PMID: 19437303

[6] Sandbank M Bottema-Beutel K Crowley S Cassidy M Feldman JI Canihuante M . Intervention effects on language in children with autism: a Project AIM meta-analysis. J Speech Lang Hear Res. (2020) 63:1537–60. doi: 10.1044/2020_JSLHR-19-00167, PMID: 32384865 PMC7842122

[7] Won H Mah W Kim E . Autism spectrum disorder causes, mechanisms, and treatments: focus on neuronal synapses. Front Mol Neurosci. (2013) 6:19. doi: 10.3389/fnmol.2013.00019, PMID: 23935565 PMC3733014

[8] McCracken JT McGough J Shah B Cronin P Hong D Aman MG . Risperidone in children with autism and serious behavioral problems. N Engl J Med. (2002) 347:314–21. doi: 10.1056/NEJMoa013171, PMID: 12151468

[9] Lavelle TA Weinstein MC Newhouse JP Munir K Kuhlthau KA Prosser LA . Economic burden of childhood autism spectrum disorders. Pediatrics. (2014) 133:e520–9. doi: 10.1542/peds.2013-0763, PMID: 24515505 PMC7034397

[10] Wong VCN . Use of complementary and alternative medicine (CAM) in autism spectrum disorder (ASD): comparison of Chinese and Western culture (Part A). J Autism Dev Disord. (2009) 39:454–63. doi: 10.1007/s10803-008-0644-4, PMID: 18784992

[11] Gold L Ayers D Bertino J Bock C Bock A Brody EN . Aptamer-based multiplexed proteomic technology for biomarker discovery. PloS One. (2010) 5:e15004. doi: 10.1371/journal.pone.0015004, PMID: 21165148 PMC3000457

[12] Nicholson JK Connelly J Lindon JC Holmes E . Metabonomics: a platform for studying drug toxicity and gene function. Nat Rev Drug Discov. (2002) 1:153–61. doi: 10.1038/nrd728, PMID: 12120097

[13] Cheng J Lan W Zheng G Gao X . Metabolomics: a high-throughput platform for metabolite profile exploration. In: Huang T , editor. Computational Systems Biology, vol. 1754 . Springer, New York (NY (2018). p. 265–92. doi: 10.1007/978-1-4939-7717-8_16, PMID: 29536449

[14] Lim S . WHO standard acupuncture point locations. Evid Based Complement Alternat Med. (2010) 7:167–8. doi: 10.1093/ecam/nep006, PMID: 19204011 PMC2862941

[15] You HZ Zhou YF Yu PB Xie J Chen J Li JJ . The efficacy of acupuncture on tic disorders in children: a retrospective and propensity score−matched study. Front Pediatr. (2021) 9:745212. doi: 10.3389/fped.2021.745212, PMID: 34805042 PMC8600324

[16] Zhang SJ Lin D Lin LL Qi SY Gong M Li SB . The use of goal attainment scaling in the acupuncture of children with intellectual disability. World J Trad Chin Med. (2022) 8:522−529. doi: 10.4103/2311-8571.351509

[17] Zhang H Liu T Zhang Z Payne SH Zhang B McDermott JE . Integrated proteogenomic characterization of human high-grade serous ovarian cancer. Cell. (2016) 166:755–65. doi: 10.1016/j.cell.2016.05.069, PMID: 27372738 PMC4967013

[18] Huang DW Sherman BT Lempicki RA . Bioinformatics enrichment tools: paths toward the comprehensive functional analysis of large gene lists. Nucleic Acids Res. (2009) 37:1–13. doi: 10.1093/nar/gkn923, PMID: 19033363 PMC2615629

[19] Szklarczyk D Gable AL Lyon D Junge A Wyder S Huerta-Cepas J . STRING v11: protein–protein association networks with increased coverage, supporting functional discovery in genome-wide experimental datasets. Nucleic Acids Res. (2019) 47:D607–13. doi: 10.1093/nar/gky1131, PMID: 30476243 PMC6323986

[20] Shen L Zhang H Lin J Gao Y Chen M Khan NU . A combined proteomics and metabolomics profiling to investigate the genetic heterogeneity of autistic children. Mol Neurobiol. (2022) 59:3529–45. doi: 10.1007/s12035-022-02768-1, PMID: 35348996

[21] Li L Ding P Lv X Xie S Li L Chen J . CD59-regulated Ras compartmentalization orchestrates antitumor T-cell immunity. Cancer Immunol Res. (2022) 10:1475–89. doi: 10.1158/2326-6066.CIR-21-1072, PMID: 36206575 PMC9716252

[22] Sanjurjo L Aran G Téllez É Amézaga N Armengol C López D . CD5L promotes M2 macrophage polarization through autophagy-mediated upregulation of ID3. Front Immunol. (2018) 9:480. doi: 10.3389/fimmu.2018.00480, PMID: 29593730 PMC5858086

[23] Tang G Gudsnuk K Kuo SH Cotrina ML Rosoklija G Sosunov A . Loss of mTOR-dependent macroautophagy causes autistic-like synaptic pruning deficits. Neuron. (2014) 83:1131–43. doi: 10.1016/j.neuron.2014.07.040, PMID: 25155956 PMC4159743

[24] Fagan K Crider A Ahmed AO Pillai A . Complement C3 expression is decreased in autism spectrum disorder subjects and contributes to behavioral deficits in rodents. Mol Neuropsychiatry. (2017) 3:19–27. doi: 10.1159/000465523, PMID: 28879198 PMC5582423

[25] Magdalon J Mansur F Teles E Silva AL De Goes VA Reiner O Sertié AL . Complement system in brain architecture and neurodevelopmental disorders. Front Neurosci. (2020) 14:23. doi: 10.3389/fnins.2020.00023, PMID: 32116493 PMC7015047

[26] Meltzer A Van De Water J . The role of the immune system in autism spectrum disorder. Neuropsychopharmacology. (2017) 42:284–98. doi: 10.1038/npp.2016.158, PMID: 27534269 PMC5143489

[27] Masi A DeMayo MM Glozier N Guastella AJ . An overview of autism spectrum disorder, heterogeneity and treatment options. Neurosci Bull. (2017) 33:183–93. doi: 10.1007/s12264-017-0100-y, PMID: 28213805 PMC5360849

[28] Gładysz D Krzywdzińska A Hozyasz KK . Immune abnormalities in autism spectrum disorder—could they hold promise for causative treatment? Mol Neurobiol. (2018) 55:6387–435. doi: 10.1007/s12035-018-1237-x, PMID: 29307081 PMC6061181

[29] Novellino F Saccà V Donato A Zaffino P Spadea MF Vismara M . Innate immunity: a common denominator between neurodegenerative and neuropsychiatric diseases. Int J Mol Sci. (2020) 21:1115. doi: 10.3390/ijms21031115, PMID: 32046139 PMC7036760

[30] Esposito G Vitagliano L Costanzo P Borrelli L Barone R Pavone L . Human aldolase A natural mutants: relationship between flexibility of the C-terminal region and enzyme function. Biochem J. (2004) 380:51–6. doi: 10.1042/BJ20031941, PMID: 14766013 PMC1224144

[31] De Vitis C Battaglia AM Pallocca M Santamaria G Mimmi MC Sacco A . ALDOC- and ENO2-driven glucose metabolism sustains 3D tumor spheroids growth regardless of nutrient environmental conditions: a multi-omics analysis. J Exp Clin Cancer Res. (2023) 42:69. doi: 10.1186/s13046-023-02641-0, PMID: 36945054 PMC10031988

[32] Naik A Thomas R AlKhalifa A Qasem H Decock J . Immunomodulatory effects of tumor lactate dehydrogenase C (LDHC) in breast cancer. Cell Commun Signal. (2025) 23:145. doi: 10.1186/s12964-025-02139-6, PMID: 40108668 PMC11924725

[33] Kasper MM Ellenbogen B Li Y Schmidt CE . Temporal characterization of hyaluronidases after peripheral nerve injury. PloS One. (2023) 18:e0289956. doi: 10.1371/journal.pone.0289956, PMID: 37616240 PMC10449126

[34] Khaliulin I Hamoudi W Amal H . The multifaceted role of mitochondria in autism spectrum disorder. Mol Psychiatry. (2025) 30:629–50. doi: 10.1038/s41380-024-02725-z, PMID: 39223276 PMC11753362

[35] Tang G Rios PG Kuo SH Akman HO Rosoklija G Tanji K . Mitochondrial abnormalities in temporal lobe of autistic brain. Neurobiol Dis. (2013) 54:349–61. doi: 10.1016/j.nbd.2013.01.006, PMID: 23333625 PMC3959772

[36] Chauhan A Gu F Essa MM Wegiel J Kaur K Brown WT . Brain region-specific deficit in mitochondrial electron transport chain complexes in children with autism. J Neurochem. (2011) 117:209–20. doi: 10.1111/j.1471-4159.2011.07189.x, PMID: 21250997 PMC4839269

[37] Carrasco M Salazar C Tiznado W Ruiz LM . Alterations of mitochondrial biology in the oral mucosa of Chilean children with autism spectrum disorder (ASD). Cells. (2019) 8:367. doi: 10.3390/cells8040367, PMID: 31018497 PMC6523430

[38] Hussain G Wang J Rasul A Anwar H Imran A Qasim M . Role of cholesterol and sphingolipids in brain development and neurological diseases. Lipids Health Dis. (2019) 18:26. doi: 10.1186/s12944-019-0965-z, PMID: 30683111 PMC6347843

[39] Wang H Liang S Wang M Gao J Sun C Wang J . Potential serum biomarkers from a metabolomics study of autism. J Psychiatry Neurosci. (2016) 41:27–37. doi: 10.1503/jpn.150057, PMID: 26395811 PMC4688025

[40] Cappuccio G Donti T Pinelli M Bernardo P Bravaccio C Elsea SH . Sphingolipid metabolism perturbations in Rett syndrome. Metabolites. (2019) 9:221. doi: 10.3390/metabo9100221, PMID: 31658741 PMC6835521

[41] Needham BD Adame MD Serena G Rose DR Preston GM Conrad MC . Plasma and fecal metabolite profiles in autism spectrum disorder. Biol Psychiatry. (2021) 89:451–62. doi: 10.1016/j.biopsych.2020.09.025, PMID: 33342544 PMC7867605

[42] Li H Zhang Z Xu Q Fu E Lyu P Pan X . Integrated transcriptomic and proteomic analyses reveal the effects of chronic benzene exposure on the central nervous system in mice. Toxicol Mech Methods. (2025) 35:101–12. doi: 10.1080/15376516.2024.2387740, PMID: 39099385

[43] O’Sharkey K Mitra S Chow T Paik SA Thompson L Su J . Prenatal exposure to criteria air pollution and traffic-related air toxics and risk of autism spectrum disorder: a population-based cohort study of California births (1990–2018) (2025). SSRN. Available online at: https://www.ssrn.com/abstract=5113444 (Accessed January 31, 2025). 10.1016/j.envint.2025.109562 40482621

[44] Dellefratte K Stingone JA Claudio L . Combined association of BTEX and material hardship on ADHD-suggestive behaviours among a nationally representative sample of US children. Paediatr Perinat Epidemiol. (2019) 33:482–9. doi: 10.1111/ppe.12594, PMID: 31657027 PMC7092642

[45] Ducker GS Rabinowitz JD . One-carbon metabolism in health and disease. Cell Metab. (2017) 25:27–42. doi: 10.1016/j.cmet.2016.08.009, PMID: 27641100 PMC5353360

[46] Tisato V Silva JA Longo G Gallo I Singh AV Milani D . Genetics and epigenetics of one-carbon metabolism pathway in autism spectrum disorder: a sex-specific brain epigenome? Genes (Basel). (2021) 12:702. doi: 10.3390/genes12050782, PMID: 34065323 PMC8161134

[47] Moat S Lang D McDowell I Clarke Z Madhavan A Lewis M . Folate, homocysteine, endothelial function and cardiovascular disease. J Nutr Biochem. (2004) 15:64–79. doi: 10.1016/j.jnutbio.2003.08.010, PMID: 14972346

[48] Bailey L Berry R . Folic acid supplementation and the occurrence of congenital heart defects, orofacial clefts, multiple births, and miscarriage. Am J Clin Nutr. (2005) 81:1213S–7S. doi: 10.1093/ajcn/81.5.1213, PMID: 15883454

[49] Djukic A . Folate-responsive neurologic diseases. Pediatr Neurol. (2007) 37:387–95. doi: 10.1016/j.pediatrneurol.2007.08.007, PMID: 18021918

[50] Raghavan R Riley AW Volk H Caruso D Hironaka L Sices L . Maternal multivitamin intake, plasma folate and vitamin B12 levels and autism spectrum disorder risk in offspring. Paediatr Perinat Epidemiol. (2018) 32:100–11. doi: 10.1111/ppe.12414, PMID: 28984369 PMC5796848

[51] Levine SZ Kodesh A Viktorin A Smith L Uher R Reichenberg A . Association of maternal use of folic acid and multivitamin supplements in the periods before and during pregnancy with the risk of autism spectrum disorder in offspring. JAMA Psychiatry. (2018) 75:176–84. doi: 10.1001/jamapsychiatry.2017.4050, PMID: 29299606 PMC5838577

[52] Surén P Roth C Bresnahan M Haugen M Hornig M Hirtz D . Association between maternal use of folic acid supplements and risk of autism spectrum disorders in children. JAMA. (2013) 309:570–7. doi: 10.1001/jama.2012.155925, PMID: 23403681 PMC3908544

[53] Schmidt RJ Iosif AM Angel EG Ozonoff S . Association of maternal prenatal vitamin use with risk for autism spectrum disorder recurrence in young siblings. JAMA Psychiatry. (2019) 76:391–8. doi: 10.1001/jamapsychiatry.2018.3901, PMID: 30810722 PMC6450282

[54] Ali A Waly MI AlFarsi YM Essa MM AlSharbati MM Deth RC . Hyperhomocysteinemia among Omani autistic children: a case-control study. Acta Biochim Pol. (2011) 58:547–51. doi: 10.18388/abp.2011_2223, PMID: 22187679

[55] Shoffner J Trommer B Thurm A Farmer C Langley WA Soskey L . CSF concentrations of 5-methyltetrahydrofolate in a cohort of young children with autism. Neurology. (2016) 86:2258–63. doi: 10.1212/WNL.0000000000002742, PMID: 27178705 PMC4909560

[56] Adams JB Audhya T Geis E Gehn E Fimbres V Pollard EL . Comprehensive nutritional and dietary intervention for autism spectrum disorder—a randomized, controlled 12-month trial. Nutrients. (2018) 10:369. doi: 10.3390/nu10030369, PMID: 29562612 PMC5872787

[57] Guo M Li L Zhang Q Chen L Dai Y Liu L . Vitamin and mineral status of children with autism spectrum disorder in Hainan Province of China: associations with symptoms. Nutr Neurosci. (2020) 23:803–10. doi: 10.1080/1028415X.2018.1558762, PMID: 30570388

[58] KaluznaCzaplinska J Michalska M Rynkowski J . Vitamin supplementation reduces the level of homocysteine in the urine of autistic children. Nutr Res. (2011) 31:318–21. doi: 10.1016/j.nutres.2011.03.007, PMID: 21530806

[59] James SJ Melnyk S Fuchs G Reid T Jernigan S Pavliv O . Efficacy of methylcobalamin and folinic acid treatment on glutathione redox status in children with autism. Am J Clin Nutr. (2009) 89:425–30. doi: 10.3945/ajcn.2008.26615, PMID: 19056591 PMC2647708

[60] DeVilbiss EA Gardner RM Newschaffer CJ Lee BK . Maternal folate status as a risk factor for autism spectrum disorders: a review of existing evidence. Br J Nutr. (2015) 114:663–72. doi: 10.1017/S0007114515002430, PMID: 26243379

[61] Poorani R Bhatt AN Dwarakanath BS Das UN . COX-2, aspirin and metabolism of arachidonic, eicosapentaenoic and docosahexaenoic acids and their physiological and clinical significance. Eur J Pharmacol. (2016) 785:116–32. doi: 10.1016/j.ejphar.2015.08.049, PMID: 26335394

[62] Mazahery H Stonehouse W Delshad M Kruger MC Conlon CA Beck KL . Relationship between long-chain n-3 polyunsaturated fatty acids and autism spectrum disorder: systematic review and meta-analysis of case-control and randomised controlled trials. Nutrients. (2017) 9:155. doi: 10.3390/nu9020155, PMID: 28218722 PMC5331586

[63] Richardson A . Long-chain polyunsaturated fatty acids in childhood developmental and psychiatric disorders. Lipids. (2004) 39:1215–22. doi: 10.1007/s11745-004-1350-z, PMID: 15736918

[64] Vancassel S Durand G Barthélémy C Lejeune B Martineau J Guilloteau D . Plasma fatty acid levels in autistic children. Prostaglandins Leukot Essent Fatty Acids. (2001) 65:1–7. doi: 10.1054/plef.2001.0270, PMID: 11487301

[65] Hirai T Umeda N Harada T Okumura A Nakayasu C OhtoNakanishi T . Arachidonic acid-derived dihydroxy fatty acids in neonatal cord blood relate symptoms of autism spectrum disorders and social adaptive functioning: Hamamatsu Birth Cohort for Mothers and Children (HBC Study). Psychiatry Clin Neurosci. (2024) 78:546–57. doi: 10.1111/pcn.13710, PMID: 39041066 PMC11488600

[66] Node K Huo Y Ruan X Yang B Spiecker M Ley K . Anti-inflammatory properties of cytochrome P450 epoxygenase-derived eicosanoids. Science. (1999) 285:1276–9. doi: 10.1126/science.285.5431.1276, PMID: 10455056 PMC2720027

[67] Kundu S Roome T Bhattacharjee A Carnevale KA Yakubenko VP Zhang R . Metabolic products of soluble epoxide hydrolase are essential for monocyte chemotaxis to MCP-1 *in vitro* and in *vivo*. J Lipid Res. (2013) 54:436–47. doi: 10.1194/jlr.M031914, PMID: 23160182 PMC3588870

[68] Wang J Fujiyoshi T Kosaka Y Raybuck JD Lattal KM Ikeda M . Inhibition of soluble epoxide hydrolase after cardiac arrest/cardiopulmonary resuscitation induces a neuroprotective phenotype in activated microglia and improves neuronal survival. J Cereb Blood Flow Metab. (2013) 33:1574–81. doi: 10.1038/jcbfm.2013.111, PMID: 23820647 PMC3790926

