# Supplementary figures and images for: Acupuncture enhances fatty acid catabolism and immune modulation in children with autism
Source: Front Psychiatry. 2025 Nov 20;16:1679154. doi: 10.3389/fpsyt.2025.1679154 (PMC12675399; doi:10.3389/fpsyt.2025.1679154)

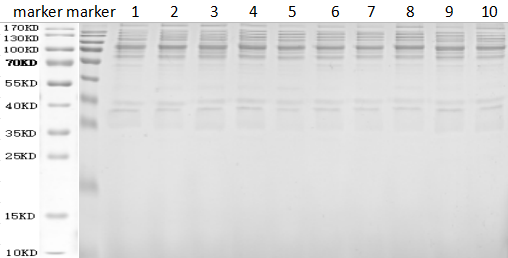

Supplement: Supplementary file 1 [file SupplementaryFile1.zip › Supplementary Figures_Gels and Blots images/Gels and Blots images_01.png]

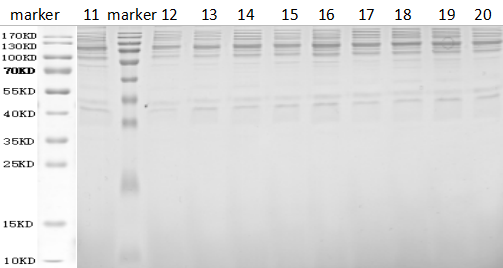

Supplement: Supplementary file 1 [file SupplementaryFile1.zip › Supplementary Figures_Gels and Blots images/Gels and Blots images_02.png]

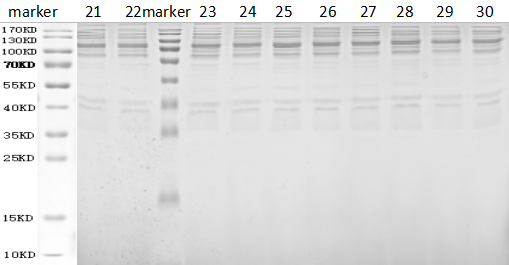

Supplement: Supplementary file 1 [file SupplementaryFile1.zip › Supplementary Figures_Gels and Blots images/Gels and Blots images_03.png]
